# 

**Published:** 1889-09

**Authors:** 


### Society Meeting—Seventh Ohio District.

The first regular meeting of the Dental Society of the Seventh District of Ohio will be held in Lincoln Club Hall, corner of Race and Seventh streets, Cincinnati, O., on Tuesday, September 17th, beginning at 10 o'clock A. M., and continuing through the day. Several papers are already promised, and from indications at hand it is confidently expected that a large number will be in attendance and a good meeting be the result.

This society was organized some months ago at Dayton, under the auspices of the Ohio State Dental Society, and it is to be hoped that at the annual meeting of the latter, this society will be able to make a good report of its organization and its work.

It is to be hoped that every dentist in the district who has the welfare of the profession at heart, will be present and be prepared to add something to the interest of the occasion. It will do us all good.

---

At one of the sessions of the Internationl Congress of Medical Jurisprudence, recently held in New York, a paper was read by Dr. C. H. Harvey describing a method of caring for the dead, consisting of treating the corpse to currents of dry air whereby the gases engendered by decomposition are carried off and burned while the body remains in a permanent state of preservation, so that it can be placed in any suitable receptacle. A company, it is said, has been formed to introduce the process in large cities.

---

The three teaching universities of Australia—Melbourne, Sidney, and Adelaide—all admit women to their lectures and degrees. It appears that there are now thirty-nine women studying in Melbourne University, twenty-three in Sidney, and thirty-four in Adelaide, the latter figures not including a number of students who are not qualifying for degrees. Adelaide first admitted women students in 1876, Melbourne and Sidney in 1881 and 1882. Ten ladies have graduated in Melbourne, nine in Sidney, and only two in Adelaide. In all three universities all prizes, scholarships, and university privileges generally are open to women who are also eligible as lecturers and professors. In Melbourne they are debarred from membership of the senate, but this seems to be the only barrier of any kind placed in their way.

### CONTENTS.

COMMUNICATIONS.

### THE DENTAL REGISTER,

**A MONTHLY MAGAZINE DEVOTED TO THE INTERESTS OF THE**

### DENTAL PROFESSION.

*Terms Reduced to* $2.00 *Per Year. Single Copies,* 25 *Cents.*

**EDITED BY PROF. J. TAFT, D.D.S.**

—AND—

**Published by SAM'L A. CROCKER & CO., Ohio Dental and Surgical Depot.**

The volume commences in January and closes in December.

The REGISTER being issued monthly is always fresh, therefore Indispensable to the practitioner who desires to keep posted up with the new inventions and improvements which are daily developed. Neither expense nor effort will be spared to give value and variety to its contents. Articles will be illustrated with well executed engravings whenever they will add to the interest or assist to explanation.

Its extensive circulation and frequency of issue make it one of the BEST DENTAL ADVERTISING MEDIUMS in the United States.

All subscriptions must begin with January or July.

Local or special notices, five lines or less, will be inserted for $3 each insertion; over five lines, 50 cts. per line.

All advertising bills payable in advance, or at furthest, after the issue of the first number containing the advertisement. All transient advertisements to be paid for in advance.

☞ CONTRIBUTIONS SOLICITED.

Exclusively Editorial Communications should be addressed to the Editor.

The latest number of the REGISTER may be ordered with or without other goods at any time, and all letters should be addressed to

**SAM'L A. CROCKER & CO., Ohio Dental and Surgical Depot, Cincinnati, O.**

